# Hot Melt Extruded Amorphous Solid Dispersion of Posaconazole with Improved Bioavailability: Investigating Drug-Polymer Miscibility with Advanced Characterisation

**DOI:** 10.1155/2014/146781

**Published:** 2014-07-21

**Authors:** Ritesh Fule, Purnima Amin

**Affiliations:** Department of Pharmaceutical Sciences and Technology, Institute of Chemical Technology, Nathalal Parekh Marg, Matunga, Mumbai, Maharashtra 400019, India

## Abstract

Invasive antifungal infections are reasons for morbidity and mortality in immunogenic patients worldwide. Posaconazole is a most promising antifungal agent against all types of invasive infections with high % of cure rate. The marketed suspension formulation has low bioavailability and is needed to be taken with food. In this paper, PCZ hot melt extruded amorphous solid dispersion (SD) with immediate release and improved bioavailability was prepared using Soluplus (Sol) as primary carrier for solubilization. Surfactants such as PEG 400, Lutrol F27, Lutrol F68, and TPGS are also used in combination with Soluplus to improve the physicochemical performance of the formulation when it comes in contact with GI (gastrointestinal) fluid. Drug-polymer miscibility of SD was investigated using advanced techniques. In the *in vivo* study, the AUC_(0–72)_ and C_max_ of PCZ/Soluplus were 11.5 and 11.74 time higher than those of pure PCZ. The formulation of the extrudate SD had an AUC_(0–72)_ and C_max_ higher than those with the commercial capsule (Noxafil). Molecular dynamic (MD) simulation studies were carried out using *in silico* molecular modelling to understand the drug-polymer intermolecular behaviour. The results of this research ensure enhanced dissolution and bioavailability of the solid dispersion of PCZ prepared by HME compared with the PCZ suspension.

## 1. Introduction

The solubility and dissolution rate of drugs relics one of the most challenging traits in formulation development. Hot melt extrusion (HME) has been established as novel strategy to produce delivery system with enhanced bioavailability as well as solubility of dissolution rate limited APIs in pharmaceutical drug development research [1–3]. This technology employs the combination of optimized parameter and temperature to formulate drug-polymer molecularly dispersed systems, which can be termed as solid dispersion (SD) or solid solution [4, 5]. The singularity and workability of the procedural features allow the development of several drug delivery systems. Also, various marketed formulations are available prepared via HME [6]. The formation of melt extrusion involves the exchange of heat energy during HME process and followed by instant cooling of the melt which affects thermodynamic and kinetic properties of forming SD apparently [7, 8]. Use of highly water soluble carrier in SD always increases the chances of crystallization due to swelling behavior when it comes in contact with the aqueous GI fluid [9]. Therefore, surface active agents or surfactants are used as inhibitors for recrystallization. HME has the unique property to maintain the amorphous state of the drug after the formation of SD and improves the solubility of the drug. Solubility of the drug substance has a significant impact on its bioavailability performance. The literature cited various methods for preparing amorphous SD such as melt method, solvent evaporation, cyclodextrin inclusion complex, and cryomilling which explained the importance of SD type of formulation strategy [10].

Posaconazole (PCZ) is newly developed extended-spectrum triazole with proven efficacy as antifungal treatment and used to treat invasive infections by* candida* species and* aspergillus* species in severely immunogenic patients. The marketed oral suspension Noxafil (40 mg/mL of PCZ concentration) should be taken with food to maximize systemic absorption. In this paper a new solid oral SD formulation has been developed using HME with improved bioavailability that can be administered without regard to food. PCZ has a very low aqueous solubility which impairs its dissolution in upper gastric fluid producing problems to prepared systems [11]. Overall, these characteristics hinder its therapeutic application by delaying the absorption rate and thereby onset of action or activity [12]. Together solubility, permeability, and dissolution rates of a drug are essential factors for determining its oral bioavailability. The literature reports generally revealed the fact that drug materials with a very low aqueous solubility will show dissolution rate limited absorption and hence poor bioavailability. Improvement of aqueous solubility in such a case is a valuable assignment to improve therapeutic efficacy [13]. PCZ is structurally analogous to itraconazole. Itraconazole has been extensively studied by various researchers comprising its SD formulations by hot melt extrusion using Soluplus as primary solubilising agent. However, there is no literature on the enhancement of solubility of PCZ by hot melt extrusion method reported. Subsequently there is a need to deliver PCZ in formulation with increased solubility and improved dissolution profile. The development of amorphous SD is a remarkable approach to increase the bioavailability of poorly soluble APIs by enhancing their rate and extent of dissolution [14].

For the current study we selected polyvinyl caprolactam-polyvinyl acetate-polyethylene glycol graft copolymer Soluplus (Sol), a novel polymer with amphiphilic properties, and explored its solubilizing potential using HME technology. The selected excipients PEG400, Lutrol F127, Lutrol F68, and TPGS were prescreened first on the basis of extrusion temperature, processing temperature, uniformity of extrudes, retention time, and solubilization. Also, these excipients contributed to the permeability of SD formulation across membrane. Various researchers reported that these excipients successfully enhance the bioavailability of poorly soluble drugs. Soluplus when processed with PCZ alone the mixing and melt formation residence time is very high which causes formation disordered sticky heterogeneous extrudes. But with addition of selected surfactant the torque decreases and extrudability and melt viscosity increase. This improved the drug distribution, uniformity, stability, and physicochemical performance of obtained extrudes. Sol offers exceptional capabilities for solubilization of BCS class II and class IV drugs, with the extensive possibility of making SD by hot melt extrusion [15]. Combination effect of Sol along with surfactants such as PEG400, L F127 (LF127), L F68 (LF68), and TPGS was studied in detail in this paper.

## 2. Materials and Methods

### 2.1. Materials

PCZ was obtained as a generous gift from ABS life science Ltd., India. Sol, LF127, LF68, and TPGS were kindly donated by BASF Corporation, Mumbai, India (head office Ludwigshafen, Germany). PEG400 of analytical grade was procured from Sd. Fine Chemicals, Mumbai, India.

### 2.2. Methods

#### 2.2.1. Calculation of Solubility Parameter (*δ*), Glass Transition Temperature (*T*_*g*_), and Florey-Huggins Parameter (*χ*)

As an indicator of the drug-polymer miscibility, values of *δ* were calculated using the Hoftyzer and vanKrevelen group contribution method described by the following equation:

(1) $$\begin{array}{c} \delta^{2}=\delta_{d}^{2}+\delta_{p}^{2}+\delta_{h}^{2}, \end{array}$$

where

(2) $$\begin{array}{c} \delta_{d}=\frac{\sum F_{di}}{V},\;\;\delta_{p}=\frac{{\left(\sum F_{pi}^{2}\right)}^{1/2}}{V},\;\;\delta_{h}={\left(\frac{\sum E_{hi}}{V}\right)}^{1/2}. \end{array}$$

Here *i* is the groups within the molecule, *δ* is the total solubility parameter, *δ*_*d*_ is the contribution from dispersion forces, *δ*_*p*_ is the contribution from polar interactions, *δ*_*h*_ is the contribution of hydrogen bonding, *F*_*di*_ is the molar attraction constant due to molar dispersion forces, *F*_*pi*_ is the molar attraction constant due to molar polarization forces, *E*_*hi*_ is the hydrogen bonding energy, and *V* is the molar volume. The solubility parameters of polymer and surfactant combinations were calculated using the following equation:

(3) $$\begin{array}{c} \delta_{1,2}=V_{1}^{\prime }\delta_{1}+V_{2}^{\prime }\delta \rho_{2}, \end{array}$$

where *V*′ is the volume fraction of each compound.

Miscibility of the drug with the polymer can be assessed based on the shift in melting endotherm or *T*_*g*_ of the drug or can be predicted theoretically using Gordon-Taylor equation based on the *T*_*g*_, densities, and weight fractions of the components. Consider

(4) $$\begin{array}{c} T_{g\;\text{mix}\;(\text{HME}\;\text{system})}=W_{1}T_{g1}+\frac{KW_{2}T_{g2}}{W_{1}}+KW_{2},\\ K\approx \frac{T_{g1}\rho_{1}}{T_{g2}\rho_{2}}, \end{array}$$

where *T*_*g*1_ is the glass transition temperature of drug, *W*_1_ and *W*_2_ are the weight fractions of the components, and *K* is the parameter calculated from the true densities (*ρ*_1_ of drug and *ρ*_2_ of polymer) and *T*_*g*2_ of the amorphous components [16]. The true density measurement of the PCZ and polymers was determined in duplicate using a gas displacement pycnometer (Accupyc 1330; Micromeritics, Norcross, Georgia).

The Flory-Huggins (FH) interaction parameter (*χ*) was calculated using the following equation:

(5) 1Tmmix−1Tmpure =−RΔHf{ln⁡⁡Φdrug+(1−1m)Φpolymer+χΦpolymer2},

where *T*_*m*_ mix is the melting temperature of the drug in the presence of the polymer, *T*_*m*_ pure is the melting temperature of the drug in the absence of the polymer, Δ*H*_*f*_ is the heat of fusion of the pure drug, *m* is the ratio of the volume of the polymer to PCZ, and Φ_drug_ and Φ_polymer_ are the volume fractions of the drug and the polymer, respectively [17].

### 2.3. Development of SD Using HME

Uniform mixing of drug with the polymer-surfactant in 1 : 1, 1 : 2, and 1 : 3 ratios was executed in a mortar-pestle to prepare physical mixtures (PMs) of 60 g each. The PMs were then transferred to a turbula mixer and further mixed for 15 min to ensure homogenous mixing. Hot melt extrusion was performed by using lab scale single screw extruder fabricated by S.B. Panchal pvt. Ltd. (Mumbai, India). Thermal parameters and speed of extruder were kept constant throughout the process for respective samples. The mixture takes about 3–5 minutes to form molten mass between walls of the screw and extruder barrel. Residence time was about 8–15 minutes for extruded to come out for various samples. The hot melt extrudates were collected, allowed to cool, milled using mixer grinder, and screened through sieve of mesh number 100. The powdered SD were stored in 50 cc amber colored glass bottles in a desiccator at room temperature and 0% humidity for further analysis and characterisation. Overall 12 optimized formulation batches were practically carried out having drug to polymer ratio 1 : 1, 1 : 2, and 1 : 3. In current paper, prepared SD formulations containing drug to polymer in 1 : 1 ratio has been discussed. Physicochemical, analytical and* in vivo* performance of the same is reported as SD (1 : 1 in ratio) has 50 % drug loading [18].

### 2.4. Physical State Characterization

#### 2.4.1. Differential Scanning Calorimetry (DSC) and (M-DSC)

Thermal analysis was performed by using PYRIS-1 Differential Scanning Calorimeter (DSC) (Perkin Elmer, USA) equipped with a liquid nitrogen attachment to study the drug and SD crystalline variability. During M-DSC accurately weighed samples (4-5 mg) were placed in sealed aluminum pans and a heat-cool-heat cycle applied involving heating from 40 to 260°C at 10°C/min then rapidly cooling to 40°C and then reheating to 260°C at 10°C/min.

#### 2.4.2. Powder X-Ray Diffractometry (PXRD)

X-ray diffraction patterns were recorded using Advance D8 system with CuK*α* radiation instrument (Bruker, USA). The recording spectral range was set at 0–60° (2*θ*) using the Cu-target X-ray tube and Xe-filled detector.

#### 2.4.3. FT-IR Spectroscopy

Pure PCZ and SD were analysed by using a Fourier transform infrared spectrophotometer model 4100 (Spectrum GX-FT-IR, Perkin Elmer, USA).

#### 2.4.4. Scanning Electron Microscopy (SEM)

The shape and surface morphology of the crystalline PCZ and PCZ-loaded SD were examined using XL 30 Model JEOL 6800 scanning electron microscope made in Japan during analysis.

#### 2.4.5. Raman Spectroscopic Analyses

The Raman spectra of the SD were recorded with a LabRamHR800 (Horiba Jovan Yvon) equipped with a 633 nm Ar-Ne laser. The laser excitation was focused using 50 objectives (Olympus Corporation) and the scattered light was totally transmitted through the notch filter towards the confocal hole and entrance slit of the spectrograph [19].

#### 2.4.6. Atomic Force Microscopy (AFM) Characterization

JXA-8530F Hyper Probe Electron Probe Microanalyzer instrument by JEOL was employed for AFM analyses. Freshly fractured extrudates on microscopic glass slides were mounted on the micrometre positioning stage of a Dimension Icon AFM with accelerating voltage of 1–30 kV. Probe current range was kept between 10 pA and 200 pA and back scattered electron images were obtained [20].

#### 2.4.7. Molecular Modelling Studies

The monomer unit structures of polymer Sol, LF127, LF68, TPGS, and PCZ were constructed by using Gaussian programme in Schrodinger, maestro software programme, USA. The energy minimization, docking, and MD-simulation studies of different conformations of drug-polymer were run to understand the structural interaction and to identify most stable conformation of drug with polymer [21, 22].

### 2.5. HPLC Analyses

PCZ and SD contents were determined using a Binary HPLC pump and 2998 UV Array detector (Agilent Corporation, Milford, Massachusetts). The formulation codes are given as PZ1, PZ2, PZ3, and PZ4 (see Table 1 for detailes). Mobile phase composed of methanol-water (75 : 25, v/v), at 1.0 mL min^−1^ flow rate, was used. The injection volume was 20 *μ*L and detection was at 262 nm for PCZ and SD [23, 24].

### 2.6. *In Vitro* Dissolution Studies

Quantity equivalent to 100 mg of PCZ was weighed from powdered SD filled inside the hard gelatine capsule that was used for the dissolution studies. The PCZ SD and marketed suspension Noxafil were investigated for their dissolution behavior. Dissolution studies were performed as per USFDA reported method, in the 900 mL with 0.3% SLS medium (sodium lauryl sulphate) at 37 ± 0.2°C using a paddle USP type II apparatus (Electrolab DBK, Mumbai, India) at speed of 25 rpm. PCZ released from the SD and Noxafil was characterised by UV absorbance measurement at a wavelength of 262 nm [25].

### 2.7. *In Vivo* Pharmacokinetics after Oral Administration

#### 2.7.1. Animals and Dosing Protocol

A single dose bioavailability study was designed in Wistar rats under fasting conditions. The animal experiments were implemented by the guidelines of the Committee for the Purpose of Control and Supervision of Experimental Animals (CPCSEA), India. The experimental protocol was approved by Institutional Animal Ethics Committee (IAEC) of Institute of Chemical Technology (Protocol number ICT/IAEC/2013/P02). Healthy male Wistar rats (weighing 170–200 g) (procured from Haffkine Institute, Mumbai, India) were used for the study. The animals were randomly divided into four groups (*n* = 6), namely, pure PCZ, Noxafil, PZ1-SD, and PZ2-SD. All rats received oral doses of 10 mg/kg including PCZ pure, marketed Noxafil suspension, and PCZ SD powder. The formulations were dispersed in pure water and then immediately administered to the rats by oral gavage according to the concentration of PCZ in the formulations. Six animals were used for each formulation. Blood samples (~0.5 mL) were drawn by retroorbital venous plexus puncture at 15 and 30 min and 1, 2, 4, 6, 8, 12, 24, 36, 48, and 72 h following oral administration after dose. Aliquots of 0.5 mL of blood were withdrawn in micro centrifuge tube previously containing acid-citrate-dextrose (ACD) buffer (0.2 mL) as an anticoagulant. The plasma blood samples were centrifuged under refrigeration at 7000 rpm for 10 min and stored at −20°C until further analyzed by HPLC. The animal study was carried out in accordance with the Code of Ethics of the World Medical Association (Declaration of Helsinki).

#### 2.7.2. HPLC Quantification of PCZ in Plasma Samples

HPLC method was developed for the analysis of PCZ in plasma of rats, a species used for safety evaluation. The HPLC analysis involved protein precipitation with methanol followed by separation on column and quantitation by UV absorbance at 262 nm. The method was sensitive with a limit of quantification of 0.25 *μ*g/mL in rat plasma. Calibration curves were used for the conversion of the PCZ/PCZ chromatographic area to the concentration of PCZ. Calibrator and quality control samples were prepared by adding appropriate volumes of standard PCZ solution in acetonitrile to drug free plasma. Calibration curves ranged from 6.4 to 36.6 *μ*g/mL (*r* ^2^ = 0.997). Briefly, an aliquot (100 *μ*L) of plasma sample was mixed with 50 *μ*L of IS solution (Itraconazole 100 *μ*g/mL) and 50 *μ*L of drug solution. After vortexing for 30 sec a protein precipitating agent methanol (500 *μ*L) was added and vortexed for 1 min. The mixture was centrifuged (Eltek TC 4100 D 232 centrifuge) at 7000 rpm for 15 min. The supernatant was filtered through 0.22 *μ*m syringe driven membrane filter unit and 50 *μ*L of the filtrate was injected onto the HPLC system. The mobile phase consisted of 0.09 M ammonium phosphate monobasic acetonitrile-triethylamine (55 : 45 v/v) and was delivered at 1 mL/min.

#### 2.7.3. Pharmacokinetic Data Analysis

The pharmacokinetic analysis of plasma concentration-time data was analyzed by one compartmental model, using Kinetica software (Thermo Scientific). Required pharmacokinetics parameters such as total area under the curve AUC_(0−72)_, half-life (*t*_1/2_), peak plasma concentration (*C*_max⁡_), and time to reach the maximum plasma concentration (*T*_max⁡_) were determined.

### 2.8. Contact Angle Evaluation Studies

The contact angle measurement was performed using G10 Contact angle meter (Kruss, Germany) to evaluate the wetting property of prepared system compared to pure hydrophobic drug PCZ. A solid disc with flat surface (100 mg) was prepared of pure PCZ and the optimized SD PZ1, PZ2, PZ3, and PZ4. A drop of distilled water was dropped on the disc surface and contact angle was measured immediately and after 60 sec of equilibrium. Three samples were measured from each batch.

### 2.9. Stability of Prepared SD

Prepared SD were kept inside the closed glass vials under controlled temperature environment inside stability chamber (Thermo Lab, India) with relative humidity of 35%, 60%, and 75% RH and temperature 37°C, 40°C, and 60°C for stability studies. Samples were removed after 1, 3, and 6 months, evaluated for dissolution rate study, and compared with those SD tested immediately after preparation [26]. The assay of the drug and SD was evaluated using HPLC at *λ* = 262 nm.

## 3. Results and Discussion

### 3.1. Mathematical Calculations

If Δ*δ* is less than 7 MPa^1/2^ both components are miscible. When Δ*δ* value was ≥10 MPa^1/2^, incompatibility and phase separation between drug and polymer occurs. The calculated solubility parameters are presented in Table 2. The calculated differences between *δ* (i.e., Δ*δ*) values of PCZ and Sol-surfactant blend were found to be in the range 2.67–4.5 MPa^1/2^, being less than 7 MPa^1/2^ indicating likely miscibility. The results suggest that Δ*δ* values are useful parameters in predicting miscibility and polymer selection for HME process [27]. Incomplete miscibility or reduced solubility can result in the formation of concentrated drug spheres that may lead to recrystallization after production and during stability [28]. From the results obtained theoretically by Gordon-Taylor analysis is found to be in similar range of experimental *T*_*g*_ (temperature used for HME process) as shown in Table 3. From the results, calculated value of FH interaction factor (*χ*) is not ≥0.5/M which signifies higher favorable extent of drug-polymer interactions at micro level. [29]. Adhesive interaction between drug and polymer favoured by the reduction in the *T*_*g*_ of SD systems which implicate the miscibility of drug and polymer is shown in Table 4.

### 3.2. Solid State Characterization

#### 3.2.1. Thermal Investigation Using DSC, MDSC, and XRD

**Figure 1:**
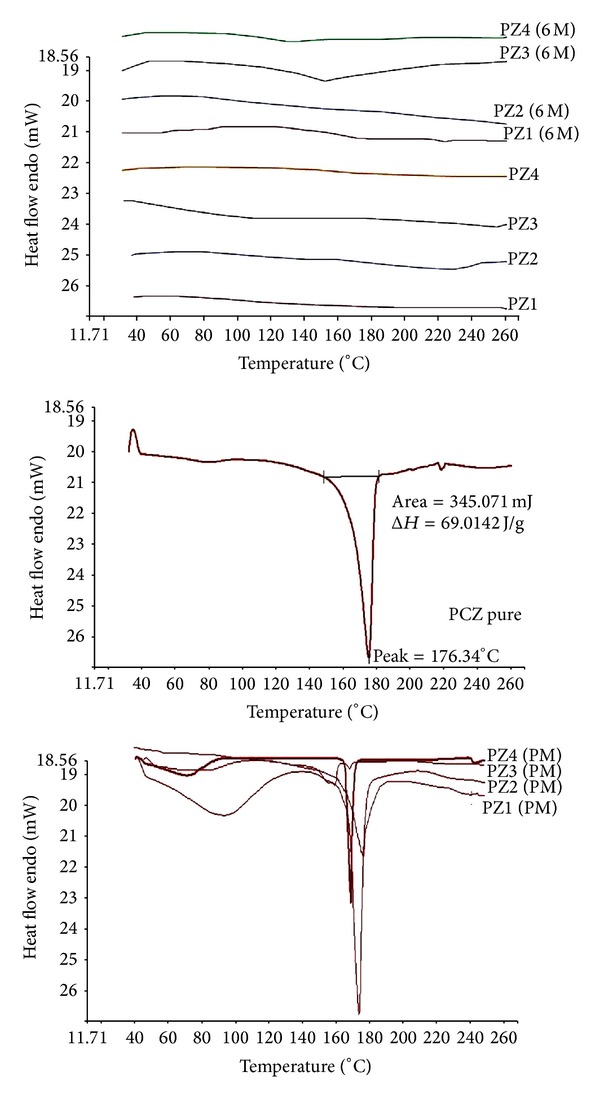
DSC pattern of SD (fresh), SD (6 months), PM, and PCZ.

**Figure 2:**
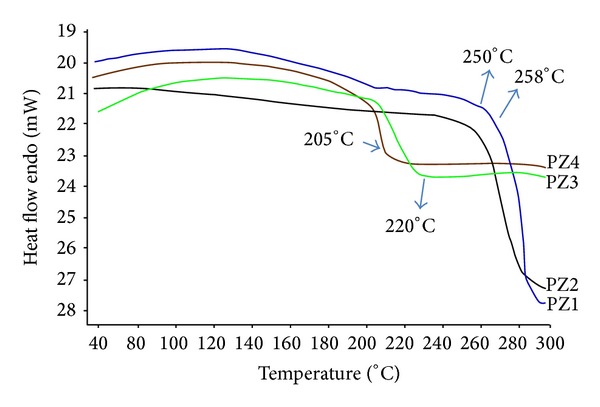
MDSC pattern of SD (6 months).

Solid-state extruded SD was analysed using DSC and MDSC. DSC was used to determine the PCZ state in the extruded SD and to identify possible drug-polymer interactions.  Figure 1 depicts the thermograms of pure PCZ which clearly show endothermic sharp peak at 176.34°C. DSC thermographs of PM show characteristic peak of crystalline PCZ. The thermograms of the hot melt extrudates SD showed different thermal behaviour for PCZ. As shown in Figure 1, the drug melting endotherms disappeared completely in case of SD. The absence of PCZ endotherms suggests either drug solubilization due to the presence of used excipients or being present in an amorphous state. Thus, it was concluded that PCZ converted to its amorphous form during hot melt processing approach. MDSC studies were performed to recognize the stable nature of amorphous SD of PCZ prepared by HME. Compared to the sharp melting peak of pure PCZ, the endothermic peaks in SD broadened during the first heating cycle and then disappeared in the second heating cycle. This is caused by gradual dissolution of the crystalline drug in the molten polymers and complete conversion to the amorphous state during the DSC heating process. MDSC shows the respective *T*_*g*_ of SD prepared approximately in similar range of temperature which signifies the amorphous drug nature in SD Figure 2. The prepared SD formulations were found to be stable in amorphous state after 6 months. The physical state of PCZ was further investigated by employing X-ray powder diffraction.

**Figure 3:**
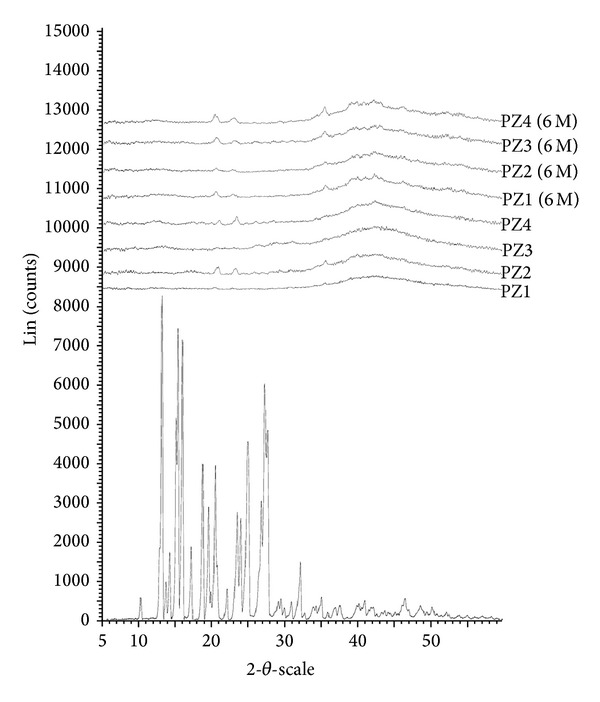
XRD pattern of pure PCZ, SD (fresh), and SD (6 months).

XRD of PCZ consist of sharp multiple peaks, indicating the crystalline nature of the drug with specific % crystallinity. In the XRD of PCZ peak intensities are observed at 10.23, 13.22, 13.82, 15.24, 16.11, 18.34, 19.27, 21.62, 22.14, 23.55, 23.64, 24.92, 26.12, 27.42, 30.86, 32.13, 35.11, and 46.23. Characteristic peaks intensities of PCZ are observed at 8100, 7500, 5500, 5300, and 4300. In the case of SD (about 2 gm), when exposed to X-ray beam, it shows disappearance of most of the crystalline endothermic peak and characteristic intensities of PCZ. This indicates complete transformation of crystalline PCZ into amorphous form during HME process. From the XRD studies of both fresh and aged SD systems amorphous nature of PCZ after HME was confirmed. The observed few intensity peaks in the diffractograms are attributed to the SD excipients such as PEG400, LF127, LF68, and TPGS as shown in Figure 3. The diffractograms indicate that PCZ is in amorphous state (or molecularly dispersed) in the SD. The XRD results are in good agreement with those of DSC thermograms.

#### 3.2.2. FTIR

**Figure 4:**
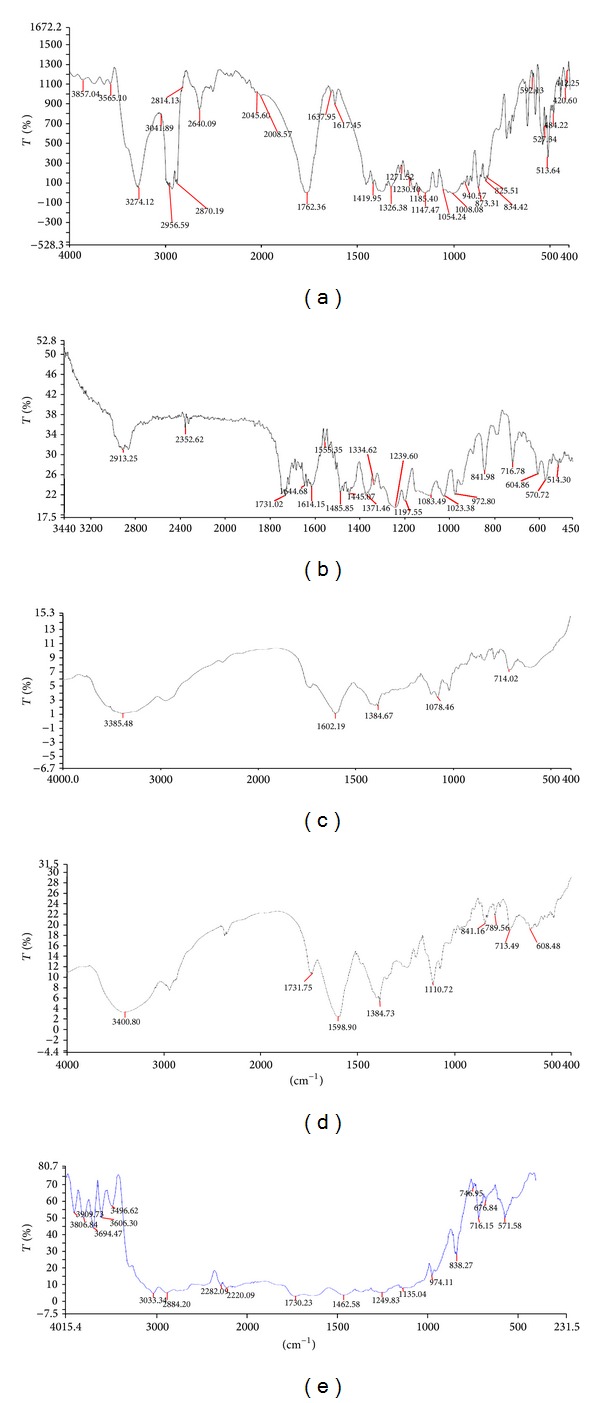
Infrared spectroscopic diagrams of pure PCZ and SD systems.

Possible interactions between drug and polymer in SD were investigated by FTIR. FTIR spectra of PCZ and SD were examined. FTIR spectrums are properly labeled and shown in Figure 4. IR of pure PCZ characteristic has sharp peaks of alkene stretching (=C–H and CH_2_) vibration at 3857.04 cm^−1^ and 3274.12 cm^−1^. Alkane stretching observed (–CH_3_, –CH_2_, and –CH) vibration at 3041.89, 2956.59, and 2870.19 cm^−1^ and also exhibited C=O stretch at 1762.36 cm^−1^ due to saturated ketone and C=O–NH stretching at 1637 cm^−1^. A selective stretching vibration at 1555.35 cm^−1^ and 1617 cm^−1^ for secondary and tertiary amine was also observed. For functional groups like C–O stretch and –C–F stretch they showed vibrations at 1054.24 cm^−1^ and 1008.08 cm^−1^, respectively. Most of the peaks that are observed in the spectral region 765–791 cm^−1^, 600–700 cm^−1^, and 820–980 cm^−1^ are due to stretching (bending =C–H and =CH_2_), –CH deformation, and –CH bending. The IR spectra of PZ1 showed characteristic peaks at 2913.25 cm^−1^, 1731.02 cm^−1^, 1535.35 cm^−1^, 1371.46 cm^−1^, 1083.40 cm^−1^, 841.98 cm^−1^, 716.78 cm^−1^, 604.86 cm^−1^, and 570.72 cm^−1^. The IR spectra of PZ2 and PZ3 showed characteristic peaks at 3385.48 cm^−1^, 1602.19 cm^−1^, 1384.67 cm^−1^, 1078.46 cm^−1^, 714.02 cm^−1^, 3400.8 cm^−1^, 1731.75 cm^−1^, 1595.9 cm^−1^, 1384.73 cm^−1^, 1110.72 cm^−1^, 841.16 cm^−1^, and 713.49 cm^−1^. The IR spectra of PZ4 showed characteristic peaks at 3033.34 cm^−1^, 2884.20 cm^−1^, 2282.09 cm^−1^, 2220.09 cm^−1^, 1730.23 cm^−1^, 1462.58 cm^−1^, 1133.04 cm^−1^, 974.11 cm^−1^, 838.27 cm^−1^, 716.15 cm^−1^, 676.84 cm^−1^, and 571.58 cm^−1^. The IR spectra of SD signify the presence of drug and no change in its functional properties. The addition of polymer and surfactant during HME process would not affect PCZ molecule stretching vibrations. Interaction between the polymer and drug in SD mixtures formed molecular dispersions with slight shifting of specific intensities compared to pure PCZ IR spectrum. The carbonyl group is more favourable for hydrogen bonding and intermolecular interactions than the nitrogen atom because of steric hindrance. For SD, the –OH stretching bands broadened and the intensity of the bands decreased to minimal, indicating specific degree of interaction between the proton donating groups of PCZ and the proton accepting groups in the Sol.

#### 3.2.3. SEM

**Figure 5:**
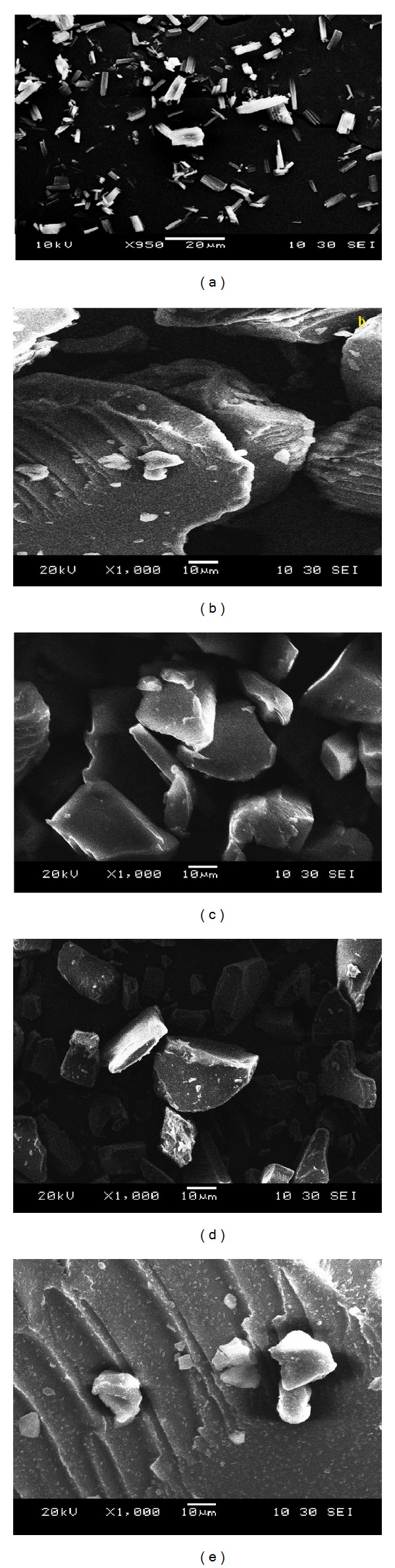
SEM images with surface morphology of pure PCZ (a), PZ1 (b), PZ2 (c), PZ3 (d), and PZ4 (e).

SEM micrograph of pure PCZ consists of large crystalline form of drug agglomerates with ordered shape and size, Figure 5(a). SEM of SD showed intrinsic miscibility of drug and polymer interface at micro level. However, in SD systems presence of relatively rough surface also suggests that hydrophilic polymer and surfactant were spread uniformly on the surface of the drug as shown in Figures 5(b), 5(c), 5(d), and 5(e). The PCZ SD appeared to be agglomerated with rough surface owing to the miscibility of drug into polymer.

#### 3.2.4. Raman Analyses

**Figure 6:**
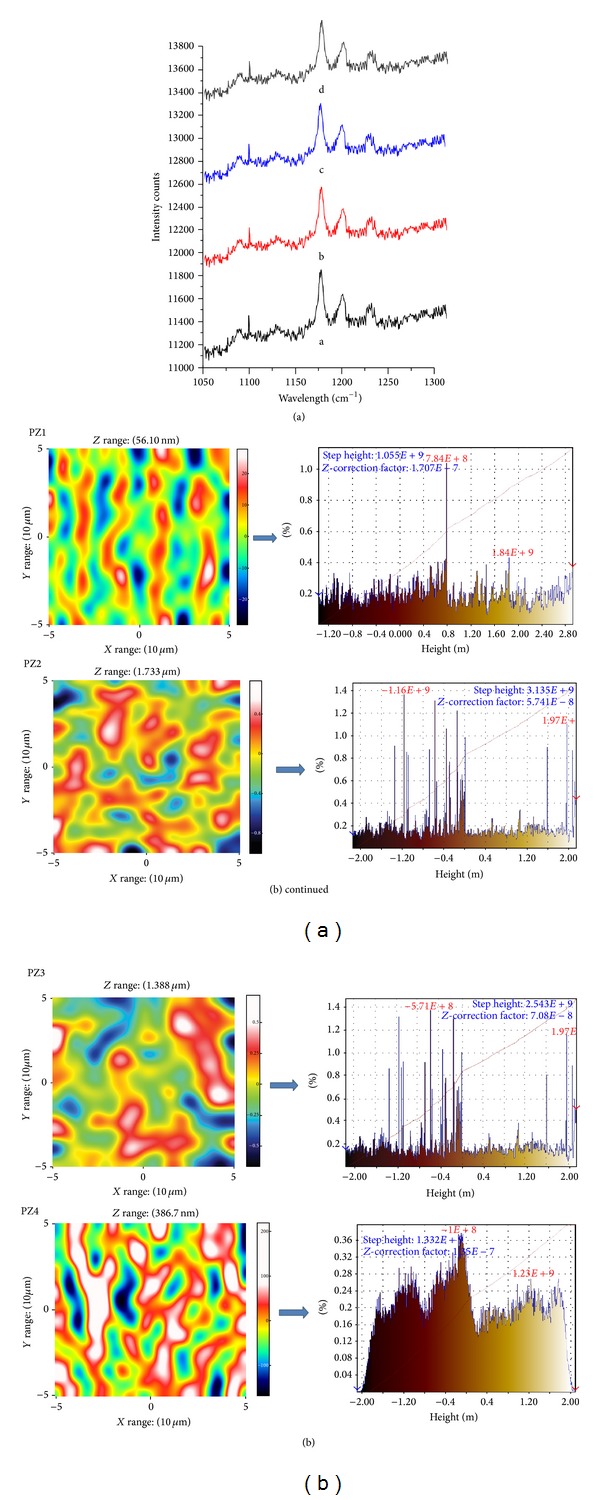
(a) Raman spectra of a (PZ1), b (PZ2), c (PZ3), and d (PZ4) SD; (b) Raman images of PZ1, PZ2, PZ3, and PZ4 SD.

With Raman spectroscopy, a laser photon is scattered by sample molecule and loses (or gains) energy during the process. The intensity of spectral features in solution is directly proportional to the concentration of the particular species as shown in Figure 6(a). Raman spectroscopy and its imaging technique are useful tools to evaluate crystal and amorphous states, including discrimination of crystalline drug in SD as shown in Figure 6(b). It confirms the presence of drug in amorphous form in SD and its uniform distribution [30]. Images of the amorphous regions in SD are described by the width at half maximum around 1150 and 1300 cm^−1^ (green area was thought to be due to amorphous drug).

#### 3.2.5. AFM Analyses

**Figure 7:**
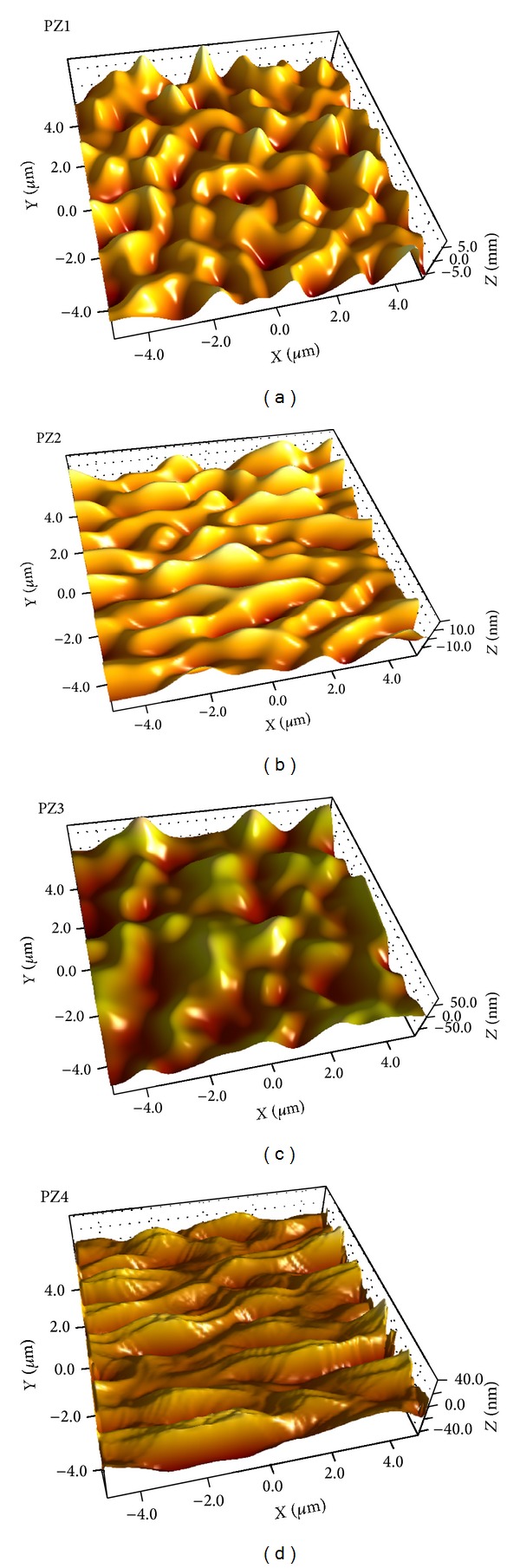
AFM microscopic image of (a) PZ1, (b) PZ2, (c) PZ3, and (d) PZ4.

Fractured fresh extrudate with smooth surfaces are used for microscopic investigations using AFM. Fracture surfaces were generated at determined fracture points on the outer surfaces of the extrudates. All extrudates had the form of transparent cylindrical rods; 2 and 3.5 cm long and of 1 cm width were selected and placed on a sheet of paper. The freshly fractured extrudates were mounted on an optical glass slide by use of a 2-component epoxy resin, which hardened within ~5 min. Before the hardening reaction had been completed the extrudate orientation was corrected to get the fracture surface as horizontal as possible. This step is mandatory to enable nondestructive imaging and automated sample changing within AFM operations [31]. Freshly fractured extrudates on microscopic glass slides were mounted on the micrometre positioning stage of a Dimension Icon AFM. Between 10 and 25 regions per sample were programmed to be automatically characterized using the software routine “programmed move” in tapping mode. Height, phase, and amplitude images were collected simultaneously, using etched silicon cantilevers with a nominal spring constant of *k* = 40 − 100 N/m (JEOL AFM Probes). The typical free vibration amplitude was in the range of *A* = 10 to 50 nm; the images were recorded with set-point amplitudes corresponding to 60–70% of the free amplitude. Image areas of 10 × 10 mm were recorded at a resolution of 1,024 × 1,024 pixels. All data were batch-processed using Scanning Probe Image Processor (SPIP 5.1.1). Height data were plane-corrected by applying a 3rd order polynomial fit [32]. Molecular fracture roughness data is also displayed in Figure 7. 3D surface AFM imaging analysis of SD formulations helps to understand the drug-polymer surface morphology as well as interaction. The roughness parameters reflect the % variation with respect to the topography mean height. It indicates from the AFM analysis that there is high level of surface interaction and amorphosization of drug inside polymer matrix observed in extrudes.

### 3.3. Molecular Dynamic Simulation Studies

**Figure 8:**
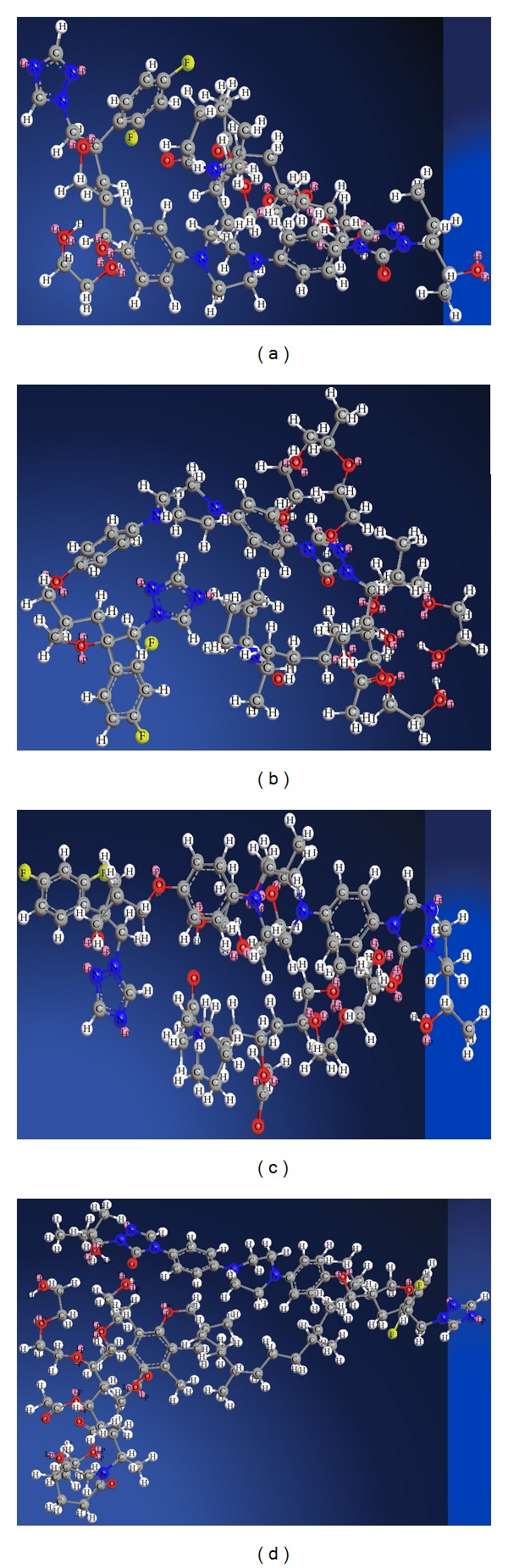
MD simulation stable conformations of PCZ-Sol-PEG400 (a), PCZ-Sol-LF127 (b), PCZ-Sol-LF68 (c), and PCZ-Sol-TPGS (d).

After energy minimization of drug and polymer strong hydrogen bonding interactions were identified. The stable conformation with lowest energy values was optimised and MD simulation dynamics was started. MD-simulation studies revealed the strong drug-polymer interactions. The effect of PEG400, LF127, LF68, and TPGS on the interaction between PCZ along with Sol was found to be favorable. The stable conformations obtained after molecular dynamic simulation showed different geometric arrangement of the molecules. The most stable was found to be Figure 8(a) with lowest energy and highest bonding interaction between drug and polymer [33, 34].

### 3.4. *In Vitro* Dissolution Studies

**Figure 9:**
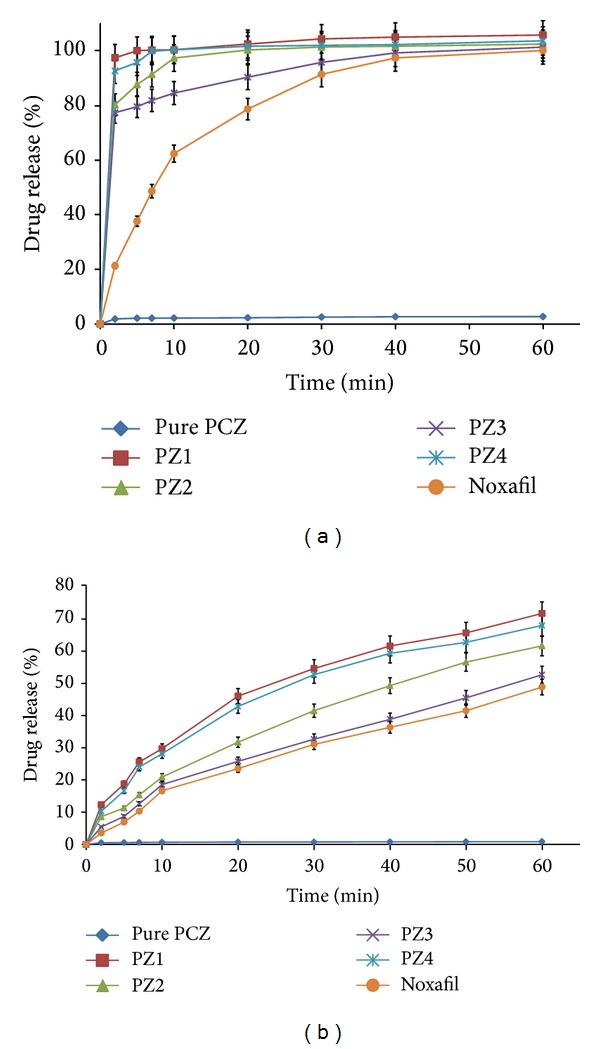
(a)* In vitro *release of SD systems in medium of 0.3% SLS (mean ± SD (*n* = 3)); (b)* in vitro* release of SD systems in distilled water (mean ± SD (*n* = 3)).

Higher apparent drug solubility and improved dissolution profiles are attributed to the amorphous nature of PCZ in SD system where PCZ is molecularly dispersed in the polymer matrix. Lattice energy of SD system is due to short range intermolecular interaction in amorphous system. When drug in SD dissolves then change in lattice energy is not destructed by the drug itself so the dissolution rate improved. Dissolution profiles of various SD are as shown in Figures 9(a) and 9(b). The dissolution of the SD with different formulations like PZ1 = 100.26%, PZ2 = 97.26%, PZ3 = 84.45%, and PZ4 = 100.25%, at the end of *T*_10_ minutes, was approximately 45.99-, 44.61-, 38.74-, and 45.98-fold higher than pure PCZ, respectively. The dissolution of the SD in water (PZ1 = 71.25%, PZ2 = 61.25%, PZ3 = 52.33%, and PZ4 = 67.55% at *T*_60_ minutes) was approximately 80.05-, 68.82-, 58.79-, and 75.89-fold than pure PCZ. The marketed suspension Noxafil dissolution studies are also carried out which are found to be inferior in terms of dissolution as compared to prepared SD. Dissolution rate of the drug in Sol-surfactant systems is governed by solubilization of the polymer to create a hydrotropic environment for the insoluble drug. It was observed that of the Sol-surfactant SD dissolved rapidly. The high dissolution rate of PCZ from the Sol-PEG400, Sol-LF127, Sol-LF68, and Sol-TPGS dispersion is believed to be due to the drug-polymer molecular intermixing at micro level. The aqueous solubility and dissolution rate of prepared SD were also significantly enhanced. In comparison to pure PCZ, the dissolution rate of physical mixtures was slightly increased probably because the hydrophilic polymer can wet the surface of drug particles and act to solubilize them. The dissolution of SD was enhanced with total release occurring within 10 min. This clearly shows that a remarkable improvement in dissolution performance was achieved by HME. From the dissolution profiles it is evident that HME processing can be employed for the manufacture of PCZ immediate release SD by processing polymer-surfactant combination. The preferred dissolution patterns can be achieved through the drug loading percentage and the extrusion process. The extrusion appeared to be an effective approach for the development of diffusion controlled SD of PCZ [35].

### 3.5. *In Vivo* Pharmacokinetic Study

**Figure 10:**
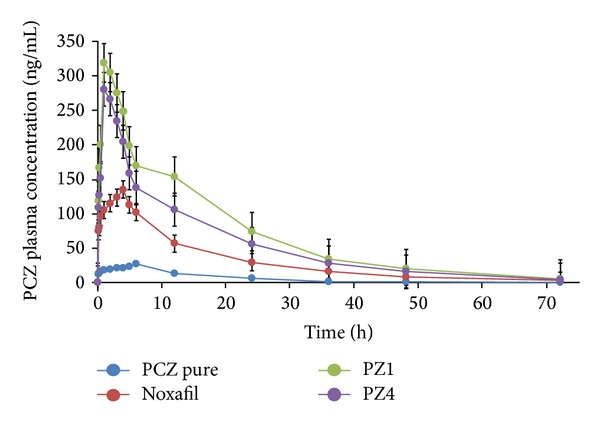
*In vivo* plasma drug concentration-time curve of PCZ, Noxafil, PZ1 SD, and PZ4 SD.

Figure 10 shows the mean plasma concentration-time curves of PCZ, after administration by oral gavage of crystalline PCZ, PCZ suspension (Noxafil), PZ1, and PZ4 SD powder to rats. The pharmacokinetic parameters are listed in Table 5. The PCZ/Sol/PEG400 (PZ1) and PCZ/Sol/TPGS (PZ4) SD had enhanced bioavailability. This was increased compared with the PCZ suspension although they all improved the bioavailability compared with pure crystalline PCZ. Also, the *T*_max⁡_ of PZ1 and PZ4 SD was prior than that of PCZ suspension. This happened due to passive transport of PCZ across the membrane in presence of Sol. This was also correlated with the dissolution profile results which claims that the dissolution rate of PZ SD was faster than that of PCZ suspension. When passing through the GI tract, the amorphous PCZ in SD was quickly dissolved and absorbed into blood circulation. PCZ primarily circulates as the parent compound in plasma. The *T*_max⁡_ of PZ1 and PZ4 SD was reached earlier at 1 h with plasma PCZ concentration of 317.97 ng/mL and 265.36 ng/mL, respectively, which indicates bioavailability enhancement. From Table 5, it can be seen that there was a significant difference between PZ1 SD and crystalline PCZ with the former having 11.6 times higher AUC_(0–72)_ than PCZ and 17.7 times higher *C*_max⁡_ than PCZ, respectively. The corresponding results for PCZ suspension were having 4.9 times higher AUC_(0–72)_ than PCZ and 6.2 times higher *C*_max⁡_ than PCZ, respectively. The significant enhancement in the bioavailability of PCZ was achieved by the PCZ/Sol SD prepared by the HME process. Also, it is reported that the food intake improves the bioavailability of PCZ. Supersaturation had a marked effect in which a high energy drug and used surfactants act as precipitation inhibitors which are induced by the gastrointestinal pH gradient. Hence, this may result in supersaturated concentrations and an increased flux across the membrane. It was also proved by the* in vitro *dissolution study that the SD showed improved dissolution, which allows further solubilization by endogenous substances like biliary fluids. Apparently, this phenomenon plays an important role in the absorption of PCZ in SD. Compared with the PCZ suspension, the higher degree of supersaturation obtained with amorphous PCZ in the SD showed a significant enhancement in oral absorption. The Soluplus and added surfactants also act as stabilizer for supersaturation upon drug release by inhibiting precipitation. These factors all increase the oral absorption and, consequently, the bioavailability of PCZ to several folds as compared to PCZ suspension and crystalline PCZ. Amorphous PCZ SD prepared using HME was found to be effective therapy with beneficial improvement in therapeutic activity of PCZ [36–38].

### 3.6. Statistical Analysis

All the results were expressed as mean ± standard deviation (SD). Statistical analysis was performed with Sigma Stat (Version 2.03) using one-way ANOVA test with *P* < 0.05 which was considered as statistically significant difference.

### 3.7. Contact Angle Calculation

The contact angle is the angle formed by the solid/liquid interface and the liquid/vapor interface measured from the side of the liquid. Contact angle also is a term used to describe a process where a drop of liquid is in contact with a solid surface and exhibits its angle towards the surface. By analyzing the contact angle, we can relate the values with the ratio and solubility and wettability of the drugs. Disc made from pure PCZ showed higher contact angle after 60 sec of equilibrium whereas SD system showed very less contact angle after equilibrium time. This effect attributed to presence of hydrophilic polymer matrix and soluble state of drug inside the system. Low contact angle value indicates highly improved wetting property of PCZ in SD system as shown in Table 6.

### 3.8. Stability on Storage

SD is thermodynamically metastable system that favours the conversion of amorphous form to the crystalline form under storage. To evaluate the physical state of the drug, the systems were characterized by XRD and DSC after storage for 6 months. The systems were stable during a 6-month period. In the case of SD, no substantial recrystallization was observed by DSC or XRD over the 6 months storage suggesting that PCZ is more stable in this formulation.

## 4. Conclusions

In the current study it was clearly demonstrated that PCZ immediate release SD formulation can be effectively produced by processing via HME with dissolution rate, solubility, and bioavailability enhancement. Novel polymer-surfactant combinations were optimised and stable SD systems were developed successfully. Utilization of Soluplus along with suitable surfactants offers excellent possibilities to develop stable amorphous SD. Selective use of surfactants with low concentrations improves process workability, increases melt viscosity with torque reduction, reduces *T*_*g*_ of blend, augments quality of extrudes, and reduces residence time of extrusion. The study revealed the importance of suitable carrier and processing technique selections are critical parameters during HME. AFM analyses revealed microscopic surface interaction between drug and polymer. MD simulation studies revealed possible molecular interaction between drug-polymer. Furthermore, this PCZ-incorporated SD gave higher dissolution compared to the commercial product and pure PCZ powder.* In vivo* bioavailability of hot melt extruded PCZ SD formulations showed several fold enhancements as compared to that of Noxafil suspension and pure PCZ. This improved therapeutically active formulation which helps to cure opportunistic invasive infections more effectively.

## Supplementary Material

The Supplementary Material is the Video of Extrusion Process of Pz1 Formulation Containing Drug and Polymer in 1:1 Ratio at Optimized Extrusion Parameters and Condition. This is Provided to Show the Morphology of Extruded Material which is in the Form of Glassy Dispersion (or Solid Solution).

## Figures and Tables

**Table 1:** HME process parameters and drug-polymer composition of prepared formulations.

Formulation codes	Batch size (gm)	Formulation composition						Extrusion temp. (°C) (speed 50 rpm for all)	Residence time (min)
		Drug (gm)	Sol (gm)	PEG400 (gm)	LF127 (gm)	LF68 (gm)	TPGS (gm)		
PZ1	60	30	29	1	—	—	—	115	7–9
PZ2	60	30	29	—	1	—	—	114	9–11
PZ3	60	30	29	—	—	1	—	118	10–12
PZ4	60	30	29	—	—	—	1	112	8–10

**Table 2:** Solubility parameter calculation for drug polymer compatibility.

Drug and polymers	*δ*_hi_	*δ*_di_	*δ*_pi_	*δ*	Δ*δ* MPa^1/2^ (not ≥7)	Drug-polymer compatibility
PCZ	9.04	3.11	15.93	18.57 (a)	—	—
Sol + PEG400	(19.43 + 9.60)/2			14.51 (b)	(a − b) = 4.06	Miscible
Sol + LF127	(19.43 + 11.20)/2			15.31 (c)	(a − c) = 3.26	Miscible
Sol + LF68	(19.43 + 12.37)/2			15.9 (d)	(a − d) = 2.67	Miscible
Sol + TPGS	(19.43 + 8.71)/2			14.07 (e)	(a − e) = 4.5	Miscible

**Table 3:** Shows the Gordon-Taylor equation calculated *T*_*g* mix_ of SD systems.

Formulation codes	*W*_1_	*T*_*g*1_	*W*_1_ ∗ *T*_*g*1_	*w*_2_	*T*_*g*2_	*W*_2_ ∗ *T*_*g*2_	*ρ*_1_	*ρ*_2_	*T*_*g*1_ ∗ *ρ*_1_	*T*_*g*2_ ∗ *ρ*_2_	*w*_1_ + *kw*_2_	*T*_*g* mix_
PZ1	0.5	176.34	88.17	2	115	230	1.3	0.86	221.13	98.9	4.971	121.16
PZ2	0.5	176.34	88.17	2	114	228	1.3	0.86	221.13	98.04	5.011	120.22
PZ4	0.5	176.34	88.17	2	118	236	1.3	0.86	221.13	101.48	4.858	124.00
PZ4	0.5	176.34	88.17	2	112	224	1.3	0.86	221.13	96.32	5.091	118.31

**Table 4:** Florey Huggins interaction parameter (*χ*) of SD.

Ratio	1/*T* mix	1/*T* pure	LHS	*R*	ΔHf	*R*/ΔHf	Φ drug	*m*	1/*m*	1 − 1/*m*	Φ Polymer	1 − 1/*m* ∗ Φ Polymer	log⁡(Φ drug)	ln⁡Φ drug + 1 − 1/*m* ∗ Φ Polymer	Φ2 Polymer	*R*/ΔHf (ln⁡Φ drug + 1 − 1/*m* ∗ Φ Polymer)	*R*/ΔHf ∗ Φ 2 Polymer *χ*	Left	*χ*
PZ1	0.008695652	0.005670863	0.003025	8.314	69.014	−0.1204683	0.5	2	0.5	0.5	1	0.5	−0.30103	0.198970004	1	−0.02396958	−0.120468311	0.00302	−0.025
PZ2	0.00877193	0.005670863	0.003101	8.314	69.014	−0.1204683	0.5	2	0.5	0.5	1	0.5	−0.30103	0.198970004	1	−0.02396958	−0.120468311	0.0031	−0.026
PZ3	0.008474576	0.005670863	0.002804	8.314	69.014	−0.1204683	0.5	2	0.5	0.5	1	0.5	−0.30103	0.198970004	1	−0.02396958	−0.120468311	0.0028	−0.023
PZ4	0.008928571	0.005670863	0.003258	8.314	69.014	−0.1204683	0.5	2	0.5	0.5	1	0.5	−0.30103	0.198970004	1	−0.02396958	−0.120468311	0.00326	−0.027

**Table 5:** *In vivo* pharmacokinetic parameters of the PCZ, Noxafil, PZ1, and PZ4 tested.

Parameters	PCZ crystalline	Noxafil	PZ1 SD	PZ4 SD
*C*_max⁡_ (ng/mL)	27.42 ± 11.26	134.74 ± 47.24	317.97 ± 14.58	265.36 ± 22.35
*T*_max⁡_ (h)	6 ± 1.22	5 ± 1.31	1 ± 1.25	1 ± 1.37
AUC_(0–72)_ (ng h/mL)	880.35 ± 124.35	4707.28 ± 245.14	10159.64 ± 1024.56	8354.28 ± 786.69
*t*_1/2_ (h)	18.24 ± 2.34	25.58 ± 3.75	25.12 ± 1.66	25.87 ± 2.44

**Table 6:** Contact angle measurement of pure PCZ and SD formulations.

Formulation codes (SD)	Contact angle (*θ*)	
	*t* = 0 sec	*t* = 60 sec
PZ1	2.5 ± 0.2	1.1 ± 0.2
PZ2	3.3 ± 0.4	1.6 ± 0.5
PZ3	3.2 ± 0.3	1.4 ± 0.4
PZ4	2.8 ± 0.5	1.2 ± 0.2
Pure PCZ	93 ± 0.8	86 ± 0.6
